# LINKS BETWEEN LONELINESS AND SLEEP AMONG PARTNERED OLDER ADULTS: AFFECTIONATE TOUCH AS A MODERATOR

**DOI:** 10.1093/geroni/igac059.3129

**Published:** 2022-12-20

**Authors:** Carly Lawrence, Christina Marini

**Affiliations:** Adelphi University, Garden City, New York, United States; Adelphi University, Garden City, New York, United States

## Abstract

Sleep problems and loneliness both increase with age and undercut older adults’ health and well-being. Loneliness is a predictor of sleep problems, however, research in this area often fails to also consider the role of relationship quality. The current study therefore focuses on spousal relationship quality as it has been shown to have a proximal influence on sleep. We examined relative effects of physical and emotional facets of relationship quality–namely affectionate touch and emotional support (in addition to loneliness). We further examined the potential for relationship quality to buffer effects of loneliness on sleep. We utilized data from a nationally representative sample of older adults from the National Social Life Health and Aging Project (NSHAP). Participants were partnered (N = 559) and completed a novel sleep module that included subjective (i.e., insomnia symptoms) and objective (i.e., Wake After Sleep Onset; WASO) markers of sleep. Upon controlling for demographics and mental and physical health, a different pattern of findings emerged for each facet of sleep. For subjective sleep, older adults who were more lonely reported more insomnia symptoms, but only when spousal emotional support was low. For objective sleep, older adults who reported more affectionate touch experienced less WASO. These findings suggest that loneliness that occurs in the context of low emotional support from spouses is particularly damaging for older adults’ subjective sleep quality. Further, affectionate touch from spouses may represent an important intervention target for promoting felt security that helps older adults stay asleep throughout the night.

